# Genetic and Phenotypic Screening of Mannose-Binding Lectin in Relation to Risk of Recurrent Vulvovaginal Infections in Women of North India: A Prospective Cohort Study

**DOI:** 10.3389/fmicb.2017.00075

**Published:** 2017-01-31

**Authors:** Namarta Kalia, Jatinder Singh, Sujata Sharma, Hardesh Arora, Manpreet Kaur

**Affiliations:** ^1^Department of Molecular Biology and Biochemistry, Guru Nanak Dev UniversityAmritsar, India; ^2^Department of Gynaecology and Obstetrics, Bebe Nanki Mother and Child Care Centre, Government Medical CollegeAmritsar, India; ^3^Department of Human Genetics, Guru Nanak Dev UniversityAmritsar, India

**Keywords:** bacterial vaginosis, mixed infections, *MBL2*, recurrent vulvovaginal infections, vulvovaginal candidiasis

## Abstract

Recurrent Vulvovaginal Infections (RVVI) is common problem associated with women of reproductive age. The function and deleterious effect of Mannose Binding Lectin 2 (*MBL2*) common polymorphisms are reported to be associated with various diseases. However, the role of *MBL2* promoter gene polymorphisms and their combined effect with structural variant along with Serum Mannose Binding Lectin (sMBL) levels in RVVI has not been investigated. The study included 258 RVVI cases and 203 age matched healthy controls. These were investigated for the distribution of *MBL2* codon 54 and promoter polymorphisms by Amplification Refractory Mutation System-Polymerase Chain Reaction (ARMS-PCR). sMBL levels were quantified by Enzyme Linked Immnosorbent Assay (ELISA). The frequency of X allele and its genotypes was significantly high in cases than controls conferring risk toward RVVI and its types (*p* < 0.05). The HXPA (OR; 2.0), LXQB (OR; 1.43) haplotypes were associated with susceptibility to RVVI cases while haplotype LYQB significantly protected against RVVI (OR; 0.58), Bacterial Vaginosis (BV) (OR; 0.27) and Mixed Infections (MI) cases (OR; 0.62) with high frequency observed in controls (*p* < 0.05). Mean sMBL levels were significantly low in RVVI, BV, Vulvovaginal Candidiasis (VVC), and MI cases compared to controls (*p* < 0.05). VVC patient showed significantly low sMBL levels than RVVI and MI cases (*p* < 0.05). The mean sMBL levels segregated based on *MBL2* genotypes and haplotypes showed significant difference in different cases groups with controls. The findings of the present study suggested that *MBL2* Y/X polymorphism and low sMBL levels were associated with susceptibility to RVVI either it is BV, VVC, or MI. Thus MBL deficiency in women with RVVI may contribute to decreased efficiency in clearing of pathogens. Hence, specific measures like administration of purified or recombinant MBL might decrease the incidence of vaginal infections recurrences and more-effective treatment.

## Introduction

Vaginal discharge is a common problem of women of reproductive age. It occurs in 1–14% of all women of reproductive age throughout the world and its prevalence in India is estimated to be 30% (Thulkar et al., 2010). It may be caused by a range of many physiological and pathological conditions including cervicitis, aerobic vaginitis, atrophic vaginitis, mucoid ectopy etc. Three common infections associated with Vulvovaginal Infections (VVI) include Bacterial Vaginosis (BV), followed by VulvoVaginal Candidiasis (VVC), and Trichomoniasis (Ryan et al., 1998; Mulu et al., 2015). About 75% of women suffer from at least one episode of VVC during their reproductive life (Sobel et al., 1998; Sobel, 2016). Moreover, a subpopulation of women has emerged with frequent complaints of vaginal infections referred as recurrent VVI (RVVI) (Powell and Nyirjesy, 2014). BV and VVC involve disturbance in normal vaginal flora which is the main cause of both infections. BV is typified by decrease in hydrogen peroxide producing lactobacilli and overgrowth of predominantly anaerobic organisms in the vagina while VVC is caused by excessive growth of Candida species, dimorphic yeast normally present in the vagina in small numbers and usually harmless (Sobel, 1988; Forsum et al., 2005). Other Risk factors for BV and VVC include black race, smoking, use of intrauterine contraceptive device, sexual activity, recent antibiotic use, pregnancy, and immunosuppression (Bleicher and Stockdale, 2015; Sobel, 2016). In contrast, Trichomoniasis is a Sexually transmitted disease. These common infections if left untreated, can result in increased risk of pelvic inflammatory disease, infertility, pre-term birth, premature rupture of membranes, vulvovaginal inflammation, and the risk of spreading other diseases (Hay et al., 1994; Sobel, 1997; Ralph et al., 1999; McClelland et al., 2007; Atashili et al., 2008; Toth et al., 2015).

Recognition of all these pathogens by innate host defense system is mediated by Pathogen recognition receptors (PRRs; Kumar et al., 2011; Santoni et al., 2015). At present, PRR families are divided into transmembrane receptors and those that reside in intracellular compartments. The former include the Toll-like receptors (TLRs) and C-type lectin receptors (CLRs), and the latter, the nucleotide-binding oligomerization domain (NOD) like receptors (NLRs), retinoic acid-inducible gene (RIG) I-like receptors (RLRs), and AIM2-like receptor (ALR) (Takeuchi and Akira, 2010; Kumar et al., 2011). Human Mannose-Binding Lectin (MBL) is a C-type-lectin that binds to a wide variety of clinical isolates of bacteria, fungi, viruses, and parasites resulting in immune responses against these pathogens (Van Emmerik et al., 1994; Neth et al., 2000; Townsend et al., 2001; Neth et al., 2002; Eisen and Minchinton, 2003; Brouwer et al., 2008; Kasperkiewicz et al., 2015). MBL is a 32 kDa Protein encoded by MBL2 mapped to 10q21.1. It is produced mainly in liver and binds to carbohydrate residues of microbes thereby triggering complement activation. It is a complex of six sets of homotrimers of a monomer containing 248 amino acids (Ezekowitz et al., 1988; Sastry et al., 1989; Taylor et al., 1989; Kurata et al., 1994). This monomer consists of four domains including a 20-amino acid N-terminal cysteine-rich domain, a collagen-like domain consisting of 18–20 tandem repeats of Gly-Xaa-Yaa, an alpha helical coiled-coil neck region, and a carbohydrate recognition domain. The neck region initiates the folding and the collagen-like region zips toward the N terminus, creating trimeric subunits. Interchain disulfide bonds link trimeric subunits to form and stabilize higher oligomers of which trimers and tetramers are probably the predominant form in circulation (Lu et al., 1990; Wallis and Drickamer, 1999; Teillet et al., 2005).

Single nucleotide polymorphisms (SNPs) present in promoter and coding region of MBL2 were documented to affect serum MBL (sMBL) levels. Three SNPs in exon 1 at codon 52 (rs5030737), 54 (rs1800450), and 57 (rs1800451), lead to the production of non-functional monomers that further interfere with the formation of higher MBL oligomers, affecting functional activity of the protein as well as its circulating levels (Sumiya et al., 1991; Lipscombe et al., 1992; Madsen et al., 1994; Terai et al., 2003; Larsen et al., 2004). In addition, three other polymorphisms in the promoter region of the MBL2, L/H (rs11003125), Y/X (rs7096206), and P/Q (rs7095891) are functionally characterized to alter the transcriptional levels significantly contributing the large variations in the promoter activity of MBL2. These findings elucidate the biological significance of these variations with respect to gene expression and hence the sMBL levels (Naito et al., 1999; Jüliger et al., 2000). Also, MBL infusion to MBL deficient cases was reported to produce satisfactory results with no adverse consequences (Valdimarsson et al., 1998; Garred et al., 2002).

A plethora of literature is available regarding the association of MBL gene variations with various diseases including Filariasis, Malaria, Tuberculosis, Trypanosomiasis, HIV infection, Systemic Lupus Erythematosus, Rheumatoid Arthritis (Meyrowitsch et al., 2010; Martiny et al., 2011; Singla et al., 2012; Weitzel et al., 2012; Li et al., 2013; Panda et al., 2013; Chen et al., 2014; Jha et al., 2014; Das and Panda, 2015). Limited reports in various populations have documented the contradicting role of codon 54 polymorphism in RVVI. Some studies have found an association of codon 54 polymorphism with increased risk of VVC (Babula et al., 2003; Liu et al., 2006; Donders et al., 2008; Wojitani et al., 2012). A single study has reported an association of codon 54 polymorphism with both VVC and BV. While, in the same study codon 57 polymorphism was found to be infrequent and not associated with disease condition (Giraldo et al., 2007). Also two studies have demonstrated a lack of association between functional polymorphisms in the first exon of *MBL2* and various RVVI (De Seta et al., 2007; Milanese et al., 2008). The above difference in association can be attributed to genetic heterogeneity between different ethnicities and thus need to be evaluated in different populations.

No studies to date have investigated the role of *MBL2* promoter gene polymorphisms and their combined effect with structural variants along with sMBL levels in RVVI and its types making the present study first approach toward it.

## Materials and methods

### Subjects

The present study enrolled 258 cases (mean age ± S.E.M, 29.33 ± 0.51 years) with symptoms including pruitis, burning, itching, soreness, pelvic pain, vaginal fishy smell, discharge, and clinically diagnosed as RVVI cases (at least four clinical episodes of VVI in the preceding 12 months) by the gynecologist. These cases were recruited from Department of Gynaecology and Obstetrics, Bebe Nanki Mother and Child Care Centre, Government Medical College, Amritsar (Punjab). The subjects with immunodeficiencies, using immunosuppressive medications or chemotherapy were excluded from the study. The control group consisted of 203 (mean age ± S.E.M, 29.33 ± 0.57 years) age-matched healthy women without any recurrent vaginal infection complaints. The study and its protocols were approved by the Institutional Human Ethics Committee of Guru Nanak Dev University, Amritsar (Punjab), India. Samples were collected after obtaining written consent of the cases and healthy controls. Details of vaginal sample collection and the diagnosis of vaginal *Candida* species, BV, and Trichomoniasis in this study population have been reported previously (Kalia et al., 2015). Vaginal samples from RVVI cases (*N* = 200) were previously processed for diagnostic test based on European (IUSTI/WHO) guidelines on the management of vaginal discharge (Sherrard et al., 2011) which categorized these 200 cases into three main groups of RVVI i.e., BV (*N* = 97), VVC (*N* = 62), and Mixed Infections (MI; *N* = 41; Kalia et al., 2015). Majority of the subjects (≈98%) had Premenopausal status. Very low history of spontaneous abortions was found in cases (4.3%) and controls (3.4%). Only 10.46% of cases were taking Oral Contraceptives Pills (OCPs) and on Intrauterine Devices (IUDs).

### Blood samples collection and processing

Blood samples were collected from the cases and healthy subjects. These samples were then subjected to DNA and serum isolation. Genomic DNA was isolated from peripheral blood mononuclear cells (PBMCs) using inorganic method (Miller et al., 1988). In brief, PBMCs were first lysed and digested, cellular proteins were then precipitated with ammonium acetate, and the DNA was precipitated with absolute ethanol. DNA samples were quantified by measuring optical density (OD) using spectrophotometer (Eppendorf, India) and yield gel electrophoresis. DNA samples with concentration above 50 ng/ml, OD > 1.5 and 260/280 ratio of 1.8 were used for genotype analysis. For serum isolation, blood samples were incubated at 37°C and centrifuged at 2000 rpm for 30 min. Isolated DNA and serum samples were stored at −80°C pending further use.

### Genotyping of *MBL2* SNPs

The three SNPs in promoters region i.e., rs11003125 (L/H), rs7096206 (Y/X), rs7095891 (P/Q), and one SNP rs1800450 (Gly54Asp) present in the first exon of the *MBL2* were genotyped using ARMS-PCR (amplification refractory mutation system-PCR). PCR was performed in 20 μl reaction volumes containing 50 ng of template DNA, 1X taq buffer containing 1.5 mM MgCl_2_, 0.025 mM of deoxynucleotide triphosphate, and 0.3 U/ml of Taq DNA polymerase (GeNei, Bangalore). Concentrations of specific primers included in each reaction are given in Table 1. All PCRs were initiated by a 10-min denaturation step at 95°C and completed by a 5-min extension step at 72°C. The PCR involves 35 cycles of 30 s at 94°C, 30 s at 55°C (62°C for codon 54), and 45 s at 72°C carried out in a thermocycler (Applied Biosystems, Life Technologies, USA). The PCR products were loaded on 1.5% agarose gel (Himedia, India) stained with ethidium bromide (SRL, India). Gel electrophoresis was carried out at 100 V and visualized using gel documentation system (Alpha imagner, USA). The genotypes were ascertained by the pattern of bands obtained from PCR products. These genotypes were also confirmed by direct sequence analysis.

**Table 1:** **Oligonucleotide primers used for *MBL2* genotyping by ARMS-PCR**.

***MBL2* SNP**	**Primer type**	**Sequence**	**Primer conc. used (p mol/μl)**	**Product size (bp)**
rs11003125	H specific forward primer	5′-GCTTACCCAGGCAAGCCTGT**G**-3′	0.1	316
	L specific forward primer	5′-GCTTACCCAGGCAAGCCTGT**C**-3′	0.1	
	Reverse common primer	5′-AACAAATGGGACCGTGCATTGC-3′	0.05	
rs7096206	Forward common primer	5′-CCTGCCAGAAAGTAGAGAGG-3′	0.15	440
	Y specific reverse primer	5′-CTGGAAGACTATAAACATGCTTTC**C**-3′	0.15	
	X specific reverse primer	5′-GGAAGACTATAAACATGCTTTC**G**-3′	0.15	
rs7095891	P specific forward primer	5′-CAGATTGTAGGACAGAGGGCATGCT**C**-3′	0.05	332
	Q specific forward primer	5′-TTGTAGGACAGAGGGCATGCT**T**-3′	0.1	
	Reverse common primer	5′-CCAGGCAGTTTCCTCTGGAAGG-3′	0.05	
rs1800450	Forward common primer	5′-CTGCACCCAGATTGTAGGACAGAG-3′	0.1	278
	A specific reverse primer	5′-CCCCCTTTTCTCCCTTGGTG**C**-3′	0.1	
	B specific reverse primer	5′-CCCCCTTTTCTCCCTTGGTG**T**-3′	0.1	

*The nucleotides defining the polymorphisms are underlined*.

### MBL quantification

The sMBL levels were quantified by enzyme linked immnosorbent assay (ELISA) kit (Ray Biotech, USA) according to manufacturer's instructions. Briefly, 100 μl of pre-diluted sample was added to microtitre plate pre-coated with anti-human MBL antibody and incubated for 2.5 h at room temperature, after washing with 1X wash buffer, biotinylated anti-human MBL antibody was added and the plate was incubated at room temperature for 1 h. Following incubation, unbound biotin conjugated anti-human antibody was removed by washing with 1X wash buffer and 100 μl streptavidin-HRP was added to all wells followed by incubation at room temperature for 45 min. After 3–4 washings, TMB substrate was added and color develops in proportion to the amount of MBL bound. The stop solution changes the color from blue to yellow; the intensity of the color was measured at 450 nm.

### Statistical analysis

CatS power calculator (http://www.sph.umich.edu) was used to calculate the sample size to achieve a power of 99% taking assumptions of 30% prevalence of vaginal discharge in India and odds ratio of 1.5 (*p* = 0.05). The demographic characteristics of cases and controls were recorded as explanatory variables and compared by Student's *t*-test. sMBL levels (mean ± S.E.M) within group were compared by one-way ANOVA followed by Tukey's multiple comparison post-test. Comparison of genotypic sMBL levels of cases with respective controls was done by Student's *t*-test. These statistical analyses were carried out by the SPSS statistical package (Version 16.0). Allelic and Genotypic frequencies of SNPs of *MBL2* were calculated by a simple allele/genotype counting method. Hardy-Weinberg equilibrium and inheritance models were determined using SNPStats (Solé et al., 2006). SNPStats association analysis is based on binary logistic regression according to the response variable [in this case, response is status (*N* = 461)]. Akaike's Information Criterion (AIC) and Bayesian Information Criterion (BIC) provided by SNPStats were used to select the best inheritance model for each specific polymorphism, with the preferred model being the one with the lowest AIC/BIC-value. Linkage disequilibrium (LD) analysis was performed with Haploview v 4.2. Allelic distribution in cases and controls was compared by odds ratio statistics using medcal software (https://www.medcalc.org/). The alleles and corresponding homozygous genotypes with major frequency in the control group have been selected as reference (OR = 1). Haplotypes were constructed from genotype data by PHASE software version 2.1.1 (Stephens et al., 2001). *p* ≤ 0.05 were considered significant.

## Results

### *MBL2* variants in RVVI cases and controls

Frequency of X allele was found to be significantly high in RVVI cases as compared to controls indicating the increased risk of RVVI (*p* < 0.0001; OR = 1.8; 95% CI = 1.36–2.37; Table 2). However, no difference in allelic frequencies of L/H, P/Q, and codon 54 polymorphism of *MBL2* was observed between RVVI cases and controls.

**Table 2:** **Distribution of allelic frequencies of *MBL2* polymorphisms in RVVI cases and controls**.

**Alleles**	**RVVI Cases (*N* = 258) Freq (%)**	**Controls (*N* = 203) Freq (%)**	**OR (95% CI)**	***p*-value**
**rs11003125**				
L(C)	285 (55.23)	236 (58.12)	1	
H(G)	231 (44.76)	170 (41.87)	1.12 (0.86–1.46)	0.37
**rs7096206**				
Y(G)	300 (58.13)	290 (71.4)	1	
X(C)	216 (41.86)	116 (28.57)	1.8 (1.36–2.37)	<0.0001^*^
**rs7095891**				
P(C)	262 (50.77)	200 (49.26)	1	
Q(T)	254 (49.22)	206 (50.73)	0.94 (0.72–1.22)	0.64
**rs1800450**				
A(G)	259 (50.19)	203 (50)	1	
B(A)	257 (49.80)	203 (50)	0.99 (0.76–1.28)	0.95

OR, odds ratio; CI, confidence intervals;

* *indicates highly significant values (P < 0.0001)*.

For the promoter Y/X variant, the frequency of YX heterozygous genotype, and XX homozygous genotype was significantly high in RVVI cases as compared to controls and shown to be associated with increased susceptibility to RVVI. However, *MBL2* SNP L/H, P/Q, and codon 54 showed no difference in genotypic frequencies between RVVI cases and controls (Table 3). All the studied genetic models, except recessive, were statistically significant for *MBL2* Y/X polymorphism. Of these, the best inheritance model was found to be dominant model with the lowest AIC/BIC-value (AIC = 611.5, BIC = 623.9) depicting that X allele carrier (either in homozygosis or heterozygosis) had a higher risk to develop RVVI than non-X carrier with (*p* < 0.0001; OR = 2.77; 95% CI = 1.87–4.09). For, P/Q polymorphism, recessive, and log additive models were the best fit model with lowest AIC/BIC value (*p* = 0.04). However, no inheritance models for L/H and codon 54 polymorphisms were found to be significant.

**Table 3:** **Distribution of genotypic frequencies of *MBL2* polymorphisms in RVVI cases and controls**.

**Genetic Models**	**Genotype**	**RVVI Cases (*N* = 258) Freq (%)**	**Controls (*N* = 203) Freq (%)**	**OR (95% CI)**	***p*-value**	**AIC**	**BIC**
**rs11003125**							
Codominant	L/L	51 (19.8%)	52 (25.6%)	1.00			
	H/L	183 (70.9%)	132 (65%)	1.41 (0.90–2.21)	0.32	638.2	654.7
	H/H	24 (9.3%)	19 (9.4%)	1.29 (0.63–2.63)	0.48		
Dominant	L/L	51 (19.8%)	52 (25.6%)	1.00	0.14	636.3	648.7
	H/L-H/H	207 (80.2%)	151 (74.4%)	1.40 (0.90–2.17)			
Recessive	L/L-H/L	234 (90.7%)	184 (90.6%)	1.00	0.98	638.5	650.9
	H/H	24 (9.3%)	19 (9.4%)	0.99 (0.53–1.87)			
Overdominant	L/L-H/H	75 (29.1%)	71 (35%)	1.00	0.18	636.7	649.1
	H/L	183 (70.9%)	132 (65%)	1.31 (0.88–1.95)			
Log-additive	–	–	–	1.21 (0.87–1.70)	0.26	637.2	649.6
**rs7096206**							
Codominant	Y/Y	69 (26.7%)	102 (50.2%)	1			
	Y/X	162 (62.8%)	86 (42.4%)	2.79 (1.86–4.17)	<0.0001^**^	613.5	630
	X/X	27 (10.5%)	15 (7.4%)	2.66 (1.32–5.36)	0.001^**^		
Dominant	Y/Y	69 (26.7%)	102 (50.2%)	1			
	Y/X-X/X	189 (73.3%)	101 (49.8%)	2.77 (1.87–4.09)	<0.0001^**^	**611.5**	**623.9**
Recessive	Y/Y-Y/X	231 (89.5%)	188 (92.6%)	1			
	X/X	27 (10.5%)	15 (7.4%)	1.47 (0.76–2.83)	0.25	637.2	649.6
Overdominant	Y/Y-X/X	96 (37.2%)	117 (57.6%)	1			
	Y/X	162 (62.8%)	86 (42.4%)	2.30 (1.58–3.35)	<0.0001^**^	619.3	631.7
Log-additive	–	–	–	2.06 (1.50–2.83)	<0.0001^**^	617.1	629.5
**rs7095891**							
Codominant	P/P	5 (1.9%)	2 (1%)	1.00			
	P/Q	252 (97.7%)	196 (96.5%)	0.52 (0.10–2.69)	0.09	635.8	652.4
	Q/Q	1 (0.4%)	5 (2.5%)	0.08 (0.01–1.19)	0.06		
Dominant	P/P	5 (1.9%)	2 (1%)	1			
	P/Q-Q/Q	253 (98.1%)	201 (99%)	0.50 (0.10–2.62)	0.4	637.8	650.2
Recessive	P/P-P/Q	257 (99.6%)	198 (97.5%)	1			
	Q/Q	1 (0.4%)	5 (2.5%)	0.15 (0.02–1.33)	0.045^*^	**634.5**	**646.9**
Overdominant	P/P-Q/Q	6 (2.3%)	7 (3.5%)	1			
	P/Q	252 (97.7%)	196 (96.5%)	1.50 (0.50–4.56)	0.47	638	650.4
Log-additive	–	–	–	0.30 (0.08–1.10)	0.048^*^	**634.6**	**647**
**rs1800450**							
Codominant	A/A	1 (0.4%)	1 (0.5%)	1.00			
	A/B	257 (99.6%)	200 (98.5%)	1.29 (0.08–20.87)	0.19	637.2	653.7
	B/B	0 (0%)	2 (1%)	(NA)			
Dominant	A/A	1 (0.4%)	1 (0.5%)	1			
	A/B-B/B	257 (99.6%)	202 (99.5%)	1.27 (0.08–20.56)	0.86	638.5	650.9
Recessive	A/A-A/B	258 (100%)	201 (99%)	1			
	B/B	0 (0%)	2 (1%)	(NA)	NA	NA	NA
Overdominant	A/A-B/B	1 (0.4%)	3 (1.5%)	1			
	A/B	257 (99.6%)	200 (98.5%)	3.88 (0.40–37.73)	0.21	636.9	649.3
Log-additive	–	–	–	0.33 (0.03–3.20)	0.3	637.4	649.8

*Akaike's Information Criterion (AIC) and Bayesian Information Criterion (BIC), Bold values indicate low AIC/BIC value*,

* *indicates (p < 0.05)*,

** *indicates (p < 0.001)*.

### *MBL2* variants in types of RVVI cases and controls

To find an association between *MBL2* polymorphisms and RVVI types, cases previously classified into BV, VVC, and MI were further compared for genotypic and allelic distribution with respect to controls (Table 4). For Y/X polymorphism, X allele was found to be significantly high in BV (*p* = 0.0006), VVC (*p* = 0.04), and MI cases (*p* = 0.003) as compared to controls. Homozygosity for X allele was significantly high in BV (*p* = 0.04) and MI (*p* = 0.004) as compared to controls but not in VVC. Heterozygosity for X allele was significantly high in BV (*p* < 0.0001) and VVC (*p* = 0.02) comparative to controls but the difference was not significant in MI.

**Table 4:** **MBL2 polymorphism, distribution, and comparison of genotypes and alleles in RVVI types with control group**.

	**No. (%) of Controls**	**No. (%) of RVVI clinical categories**			**Genotypic and allelic comparison**					
	**Controls (*N* = 203)**	**BV (*N* = 97)**	**VVC (*N* = 62)**	**MI (*N* = 41)**	**BV vs. Controls**		**VVC vs. Controls**		**MI vs. Controls**	
					**OR (95% CI)**	***p*-value**	**OR (95% CI)**	***p*-value**	**OR (95% CI)**	***p*-value**
**rs1003125**										
**Genotypes**										
LL	52 (25.61)	17 (17.5)	19 (30.64)	8 (19.51)	1		1		1	
LH	132 (65.02)	66 (68.04)	37 (59.6)	30 (73.17)	1.52 (0.82–2.84)	0.18	0.76 (0.40–1.45)	0.41	1.47 (0.63–3.43)	0.36
HH	19 (9.359)	14 (14.4)	6 (9.67)	3 (7.31)	2.25 (0.93–5.44)	0.07	0.86 (0.30–2.48)	0.78	1.02 (0.24–4.27)	0.97
**Alleles**										
L	236 (58.128)	100 (51.5)	75 (60.48)	46 (56.09)	1		1		1	
H	170 (41.87)	94 (48.4)	49 (39.51)	36 (43.90)	1.30 (0.92–1.84)	0.12	0.90 (0.60–1.36)	0.64	1.08 (0.67–1.75)	0.73
**rs7096206**										
**Genotypes**										
YY	102 (50.2)	23 (23.71)	21 (33.87)	12 (29.26)	1		1		1	
YX	86 (42.3)	65 (67.01)	35 (56.45)	21 (51.21)	3.35 (1.92–5.84)	<0.0001^***^	1.97 (1.07–3.64)	0.02^*^	2.07 (0.96–4.46)	0.06
XX	15 (7.38)	9 (9.27)	6 (9.67)	8 (19.51)	2.66 (1.03–6.82)	0.04^*^	1.94 (0.67–5.59)	0.21	4.53 (1.59–12.90)	0.004^**^
**Alleles**										
Y	290 (71.4)	111 (57.21)	77 (62.09)	45 (54.87)	1		1		1	
X	116 (28.57)	83 (42.78)	47 (37.90)	37 (90.24)	1.86 (1.30–2.67)	0.0006^***^	1.52 (1.00–2.32)	0.04^*^	2.05 (1.26–3.33)	0.003^**^
**rs7095891**										
**Genotypes**										
PP	2 (0.985)	0	1 (1.61)	4 (9.75)	–	NA	1		1	
PQ	196 (96.55)	96 (98.96)	61 (98.38)	37 (90.24)	1		0.62 (0.05–6.98)	0.7	0.09 (0.01–0.53)	0.007^**^
QQ	5 (2.46)	1 (1.03)	0	0	0.40 (0.04–3.54)	0.41	–	NA	–	NA
**Alleles**										
P	200 (49.26)	96 (49.48)	63 (50.80)	45 (54.87)	1		1		1	
Q	206 (50.73)	98 (50.51)	61 (49.19)	37 (45.12)	0.99 (0.70–1.39)	0.95	0.94 (0.62–1.40)	0.76	0.79 (0.49–1.28)	0.35
**rs1800450**										
**Genotypes**										
AA	1 (0.49)	1 (1.03)	0 (0)	0 (0)	–	NA	–	NA	–	NA
AB	200 (98.52)	96 (98.9)	62 (100)	41 (100)	–	NA	–	NA	–	NA
BB	2 (0.98)	0 (0)	0 (0)	0 (0)	–	NA	–	NA	–	NA
**Alleles**										
A	202 (49.75)	98 (50.5)	62 (50)	41 (50)	1		1		1	
B	204 (50.24)	96 (49.4)	62 (50)	41 (50)	0.97 (0.68–1.36)	0.86	0.99 (0.66–1.48)	0.96	0.99 (0.61–1.59)	0.96

*OR, Odds Ratio; CI, Confidence Intervals*,

* *indicates significant p-values (p < 0.05)*,

** *indicates high significant values (p < 0.01)*,

*** *indicates highly significant values (p < 0.001)*.

For L/H, P/Q, and codon 54 polymorphisms, no significant difference was observed either in allelic or genotypic frequency between RVVI cases types and controls except for P/Q polymorphism where Heterozygosity was found to be significantly low in MI cases than controls (*p* = 0.007). Also, comparison between RVVI types showed no significant difference in *MBL2* distribution (not shown in table). For P/Q polymorphism, no homozygote for major allele in case of BV and for minor allele in case of VVC and MI was observed. For codon 54 polymorphism, only one homozygote of wild allele A in case of BV was observed whereas no homozygote was observed in VVC and MI.

### Linkage disequilibrium and haplotypes

**Figure 1:**
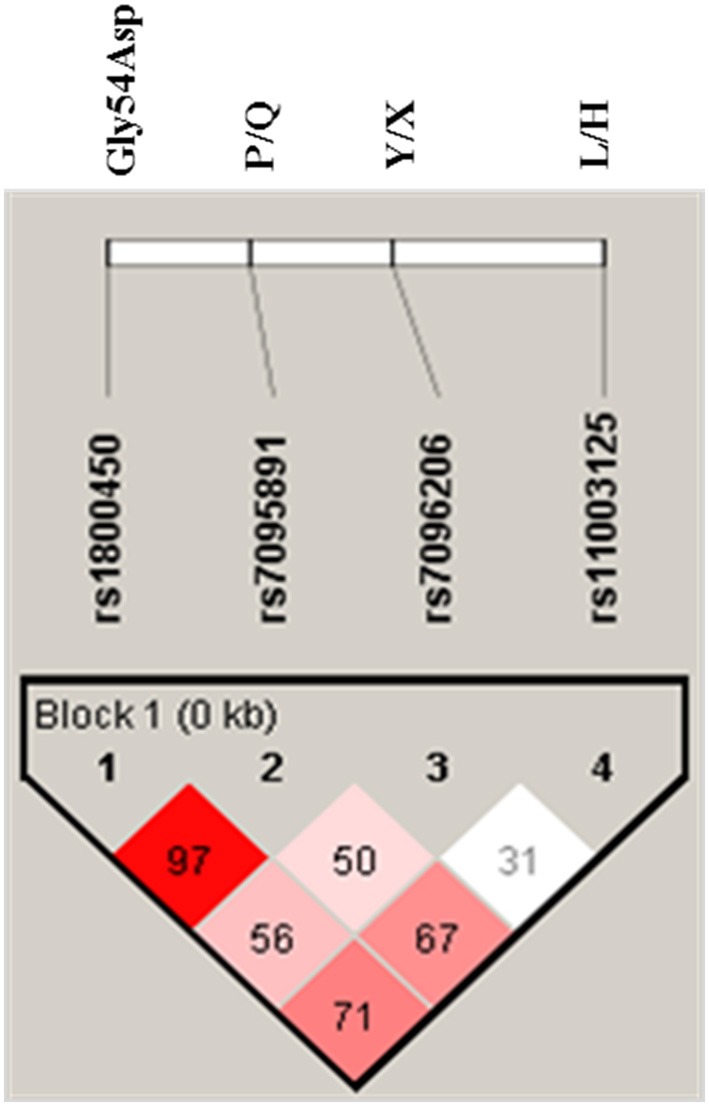
**LD pattern of *MBL2* variants in all subjects**. Numbers indicate the *D*′-value expressed as percentile.

The Linkage disequilibrium (LD) between studied SNPs indicated strong LD between codon 54 and P/Q variant (Figure 1). While others SNPs i.e., codon 54 and P/Q variants were found to be in fairly high LD with L/H and Y/X variants. The *MBL2* secretor haplotypes distributions and their comparison among cases-control groups were observed (Table 5). There were significant difference (global *p* = 0.01) in haplotype frequency between cases and controls. Apart from well-known *MBL2* secretor haplotypes, present study have also observed nine additional *MBL2* haplotypes, of which HXPA, LYQB, HYQB, LXQB, and HXQB, were observed with frequency ≥ 0.05. Independent analysis of each individual haplotypes indicated that haplotype LYQB was found to be significantly low in total RVVI cases (*p* = 0.006), BV cases (*p* < 0.0001), and MI cases (*p* = 0.05) than controls. Haplotypes, HXPA was significantly higher in RVVI cases (*p* = 0.05) and MI cases (*P* = 0.04) than controls and LXQB was significantly higher in RVVI cases (*p* = 0.04) than controls. However, no other difference was observed in haplotypes distribution.

**Table 5:** **Distribution and comparison of MBL2 secretor haplotypes in cases and controls**.

**Haplotypes**	**No. (%) of controls**	**No. (%) of cases**				**Haplotype comparison**									
		**Total RVVI Cases**	**Clinical categories of RVVI**			**RVVI vs. Controls**		**BV vs. Controls**		**VVC vs. Controls**		**MI vs. Controls**			
	**(*N* = 406)**	**(*N* = 516)**	**BV (*N* = 194)**	**VVC (*N* = 124)**	**MI (*N* = 82)**	**OR (95% CI)**	***p*-value**	**OR (95% CI)**	***p*-value**	**OR (95% CI)**	***p*-value**	**OR (95%CI)**			***p*-value**
**HIGH SECRETOR**															
HYPA	127 (31.3)	163 (31.5)	73 (37.6)	40 (32.2)	26 (31.7)	1		1		1			1		
LYPA	46 (11.2)	44 (8.43)	14 (7.21)	16 (12.9)	7 (8.5)	0.74 (0.464–1.19)	0.22	0.52 (0.27–1.02)	0.06	1.10 (0.56–2.15)	0.77		0.74 (0.30–1.82)	0.51	
LYQA	1 (0.24)	1 (0.19)	1 (0.5)	0	0	0.77 (0.04–12.57)	0.86	1.73 (0.10–28.23)	0.69	–	NA		–	NA	
HYQA^†^	1 (0.24)	1 (0.19)	1 (0.5)	0	0	0.77 (0.04–12.57)	0.86	1.73 (0.10–28.23)	0.69	–	NA		–	NA	
**LOW SECRETOR**															
LXPA	14 (3.45)	19 (3.71)	3 (1.54)	3 (2.41)	1 (1.2)	1.05 (0.51–2.19)	0.88	0.37 (0.10–1.34)	0.13	0.68 (0.18–2.48)	0.56		0.34 (0.04–2.77)	0.31	
HXPA^†^	12 (3)	31 (6.1)	6 (3.0)	3 (2.41)	7 (8.5)	2.01 (0.99–4.07)	0.05^*^	0.86 (0.31–2.41)	0.78	0.79 (0.21–2.95)	0.73		2.84 (1.02–7.92)	0.04^*^	
LYPB	1 (0.24)	2 (0.38)	0 (0)	0	2 (2.4)	1.55 (0.13–17.37)	0.71	–	NA	–	NA		–	NA	
LYQB^†^	96 (23.48)	72 (14)	15 (7.73)	19 (15.3)	9 (10.9)	0.58 (0.39–0.85)	0.006^**^	0.27 (0.14–0.50)	0.0001^***^	0.62 (0.34–1.15)	0.13		0.45 (0.20–1.02)	0.05^*^	
HYPB^†^	0	1 (0.19)	0 (0)	0	0 (0)	–	NA	–	NA	–	NA		–	NA	
HYQB^†^	19 (4.63)	16 (3.1)	7 (3.6)	2 (1.6)	1 (1.2)	0.65 (0.32–1.32)	0.24	0.64 (0.25–1.59)	0.33	0.33 (0.07–1.49)	0.15		0.25 (0.03–2.00)	0.19	
LXPB^†^	0	2 (0.38)	0 (0)	1 (0.8)	1 (1.2)	–	Na	–	NA	–	NA		–	NA	
LXQB^†^	79 (19.6)	146 (28.2)	67 (34.5)	36 (29)	26 (31.7)	1.43 (1.00–2.06)	0.04^*^	1.47 (0.95–2.27)	0.07	1.44 (0.85–2.45)	0.17		1.60 (0.87–2.96)	0.12	
HXQB^†^	10 (2.41)	17 (3.3)	7 (3.6)	4 (3.2)	1 (1.2)	1.32 (0.58–2.99)	0.4990	1.21 (0.44–3.33)	0.70	1.27 (0.37–4.27)	0.69		0.48 (0.05–3.98)	0.50	
HXPB^†^	0	1 (0.19)	0	0	1 (1.2)	–	NA	–	NA	–	NA		–	NA	
**HIGH vs. LOW SECRETOR**															
High^‡^	175 (43.1)	208 (40.3)	89 (45.8)	56 (45.1)	33 (40.2)	0.89 (0.68–1.16)	0.3929	1.11 (0.79–1.57)	0.52	1.08 (0.72–1.62)	0.68		0.88 (0.54–1.44)	0.63	
Low^§^	231 (56.8)	308 (59.6)	105 (54)	68 (54.8)	49 (59.7)	1		1		1			1		

*Global p-value for case/control haplotype association was 0.01*,

† Novel secretor haplotypes observed in this study;

‡ High secretor MBL2^*^ (HYPA_LYQA_LYPA_HYQA);

§ Low secretor MBL2^*^ (LXPA_LYPB_LYQB_HYPB_HXPA_HYQB_LXPB_LXQB_HXQB_HXPB); NA, not applicable.

* p < 0.05;

** p < 0.01;

*** *p < 0.001*.

*High and low secretor status of haplotypes was designated based on previous literature*.

### sMBL levels in RVVI cases and healthy controls

**Figure 2:**
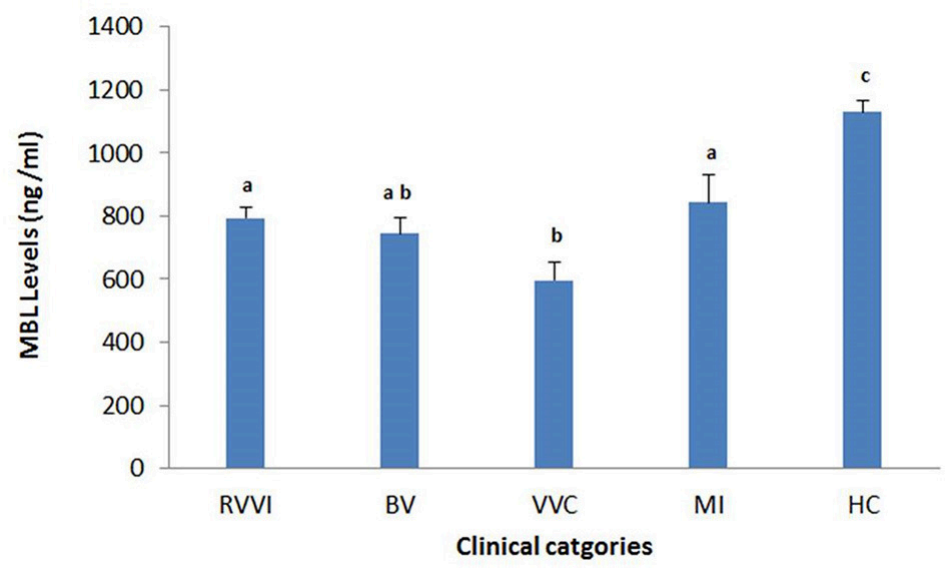
**Serum MBL levels in different clinical categories of RVVI and healthy controls (HC)**. Data are mean ± standard error, One-way ANOVA and Tukey's HSD. Means that do not share the same superscript are significantly different from each other at *P* < 0.05.

Tukey's test revealed that RVVI cases and its subtypes BV, VVC, and MI showed significantly low level of sMBL as compared to controls (Figure 2). Also, significantly low sMBL levels were observed in VVC cases as compared to RVVI and MI at *p* < 0.05 (Figure 2).

### Genotype-phenotype association of MBL polymorphisms

**Figure 3:**
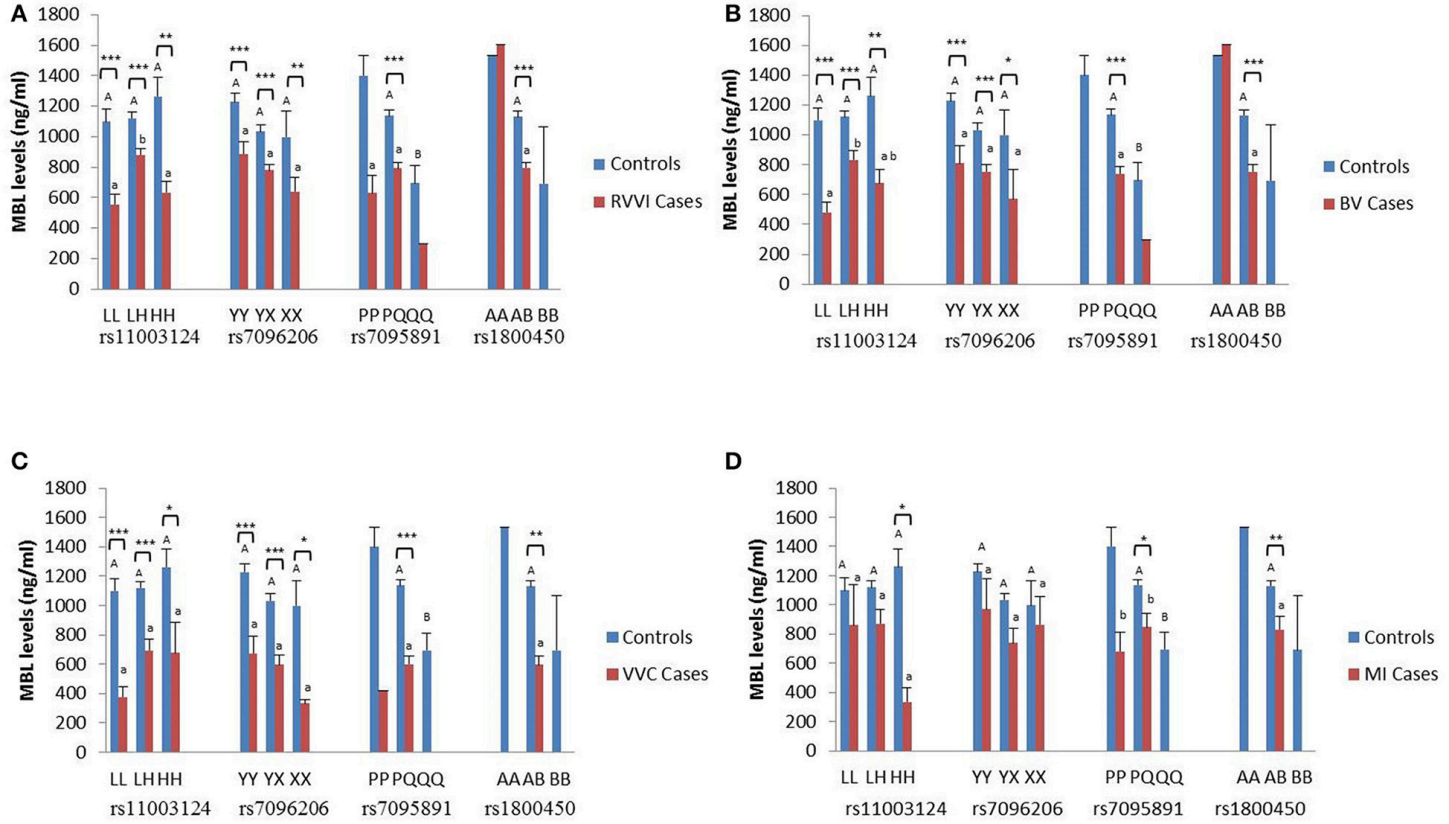
**Distribution of sMBL levels in different MBL2 genotypes**. Serum MBL levels (mean ± standard error) according to different genotypes of studied *MBL2* variants in RVVI with controls **(A)** and RVVI types with controls that includes BV **(B)**, VVC **(C)**, and MI **(D)**. Asterisks represents comparison of cases with respective controls by Student's *t*-test (^*^p < 0.05; ^**^p < 0.01; ^***^p < 0.001). Letters denote Tukey's multiple comparison test. Upper case letters denote Tukey's test within controls of one particular SNP, Lower cases letter denote Tukey's test within cases of one particular SNP. Means that do not share the same superscript are significantly different from each other at *p* < 0.05. Bars with no letters are not included in statistical analysis due to their low frequency of genotype observed in population.

The sMBL levels were segregated based on genotypes of different *MBL2* polymorphisms studied in the different cases groups and controls (Figures 3A–D). Data analysis by Tukey's test for genotypic sMBL levels in one particular SNP indicated that, within controls, variant QQ genotype contributed significantly low sMBL levels than PQ genotype. Similarly, sMBL levels for L/H polymorphism when compared within cases revealed that, in RVVI cases, LH genotype contribute significantly high sMBL levels than LL and HH genotypes while in BV cases significant difference was found between LL and LH genotypes only.

Comparison of genotypic sMBL levels of cases with respective controls by Student's *t*-test showed, significantly low sMBL levels in various L/H genotypes of RVVI, BV, and VVC cases as compared to respective control genotypes. However, in MI cases, only significant difference was observed between sMBL levels of homozygous variant genotype HH with respective genotype of control. For Y/X polymorphism, overall significant low sMBL levels were observed in various Y/X genotypes in RVVI, BV, and VVC cases as compared to respective genotypes of controls. For P/Q polymorphism, only significant difference was found in heterozygous PQ genotype of RVVI and its types with respective controls genotype. For A/B polymorphism, significant low sMBL levels of AB genotype were observed in RVVI and its types as compared to AB genotype of controls.

### Haplotypes and sMBL levels

**Figure 4:**
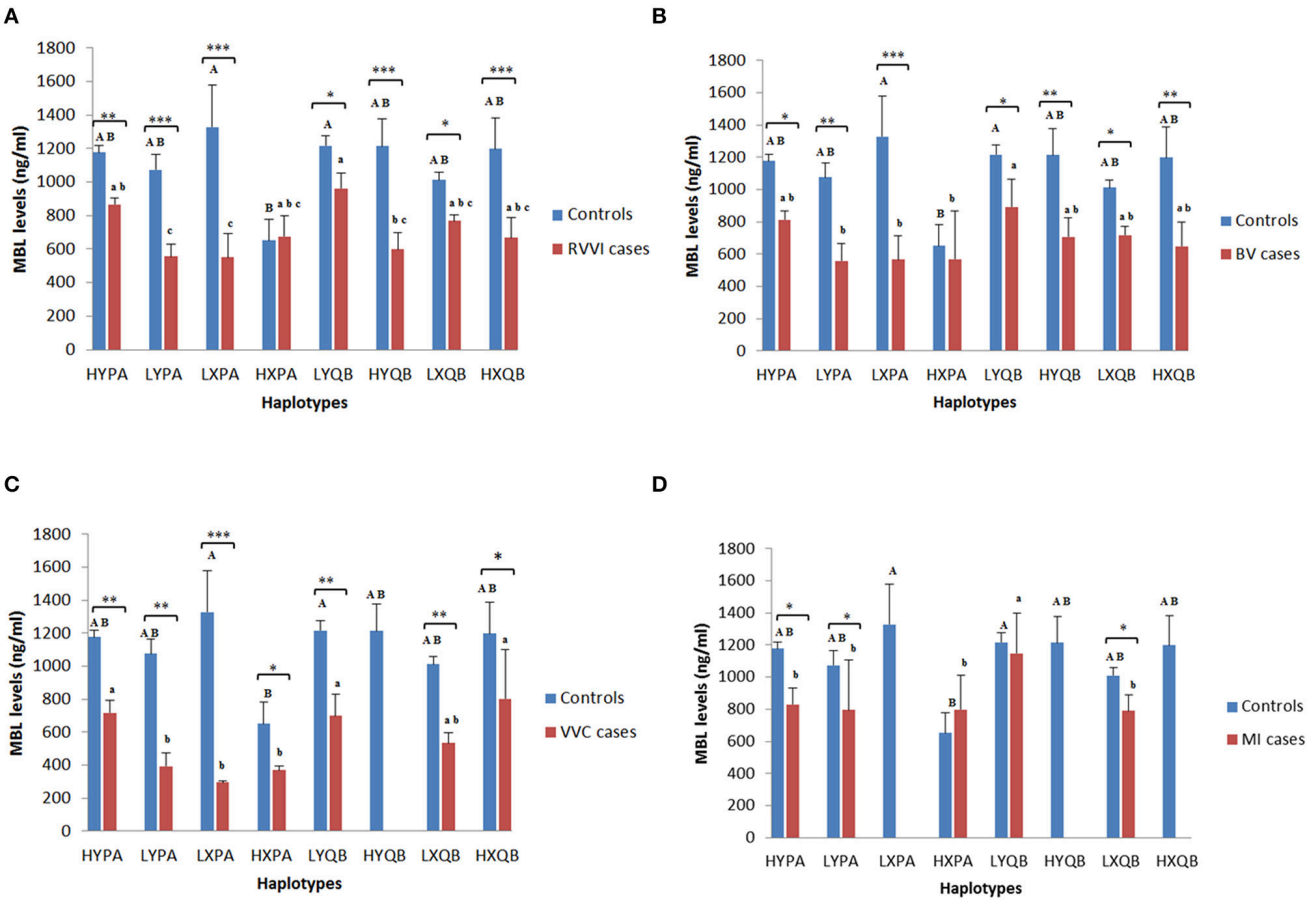
**Distribution of MBL levels in different MBL2 Haplotypes**. Serum MBL levels (mean ± standard error) according to different haplotypes of studied *MBL2* variants in RVVI with controls **(A)** and RVVI types with controls that includes BV **(B)**, VVC **(C)**, and MI **(D)**. Asterisks represents comparison of cases with respective controls by Student's *t*-test (^*^p < 0.05; ^**^p < 0.01; ^***^p < 0.001). Letters denote Tukey's multiple comparison test. Upper case letters denote Tukey's test within controls, lower cases letter denote Tukey's test within cases. Means that do not share the same superscript are significantly different from each other at *p* < 0.05.

Mean sMBL levels of Individual haplotypes in cases and control groups were also studied (Figures 4A–D). In **controls**, HXPA haplotype was contributing significant low levels than LXPA and LYQB haplotypes while no difference was observed among sMBL of other haplotypes within control. In **RVVI** cases, LYPA and LXPA haplotypes were contributing significant low levels than HYPA and LYQB haplotypes, while no differences were observed in sMBL of other haplotypes within RVVI cases. Also, all the RVVI haplotypes except HXPA haplotype were found to be contributing significant low levels than their respective haplotype in controls. In **BV** cases, LYPA, LXPA, and HXPA haplotypes were contributing significant low levels than LYQB haplotype. All the haplotypes in BV except HXPA haplotype contributed significantly low levels than respective haplotypes in controls. In **VVC** cases, LYPA, LXPA, and HXPA haplotypes were contributing significant low levels than HYPA, LYQB, and HXQB haplotypes. Significant difference was observed between all the haplotypes of VVC cases with respective controls haplotypes. In **MI** cases, haplotypes HYPA, HXPA, LYPA, and LXQB were contributing significantly low level than LYQB. However, significant difference was observed for HYPA, LYPA, and LXQB haplotypes of MI cases with respective haplotypes of controls.

## Discussion

Majority of the microorganism induced responses are triggered by a set of germ line encoded PRRs that recognize Pathogen Associated Molecular Patterns (PAMPs) present on microbial pathogen surfaces (Mogensen, 2009; Kumar et al., 2011; Santoni et al., 2015). Genetic changes in these PRRs are suggested as a contributing factor for disease development (Mogensen, 2009). MBL is one such multi functioning PRR that is involved in fine tuning the immune system. Our earlier report, based on *in silico* analysis filtered out, 12 functionally important SNPs of *MBL2* gene (Kalia et al., 2016). Out of these, four SNPs i.e., H/L, Y/X, and P/Q and codon 54 of *MBL2* are widely studied and found to be associated with different diseases. However, no studies to date have investigated the role of these variants along with its serum levels in RVVI and its types making the present study first approach toward it.

The best fit model for Y/X polymorphism depicted X allele as a dominant allele. Homozygosity and heterozygosity of X allele was found at significantly higher frequencies in RVVI cases and its types which increases the risk for developing RVVI, BV, VVC, and MI. Concluding that one leading mechanism responsible for RVVI and its types in women is MBL Y/X polymorphism. No study, to our knowledge has evaluated the role of MBL2 promoter polymorphism in association with RVVI and its types. However, these promoter variants have been associated in susceptibility toward various diseases (Meyrowitsch et al., 2010; Martiny et al., 2011; Singla et al., 2012; Weitzel et al., 2012; Li et al., 2013; Panda et al., 2013; Chen et al., 2014; Jha et al., 2014; Das and Panda, 2015).

No significant difference was found for MBL codon 54 polymorphism in RVVI cases and its types. This finding is in agreement with two studies demonstrating, lack of association between functional polymorphisms in the first exon of *MBL2* with VVC and recurrent BV (De Seta et al., 2007; Milanese et al., 2008). Also no homozygous genotype (BB) of codon 54 polymorphism was observed in RVVI cases and its types and this finding of the present study was in accordance with a previous study, where no BB genotype was observed in VVC cases (Liu et al., 2006). However, the results of the present study were contrary to some other studies where codon 54 polymorphism was found to be associated with increased risk of VVC and BV (Babula et al., 2003; Liu et al., 2006; Giraldo et al., 2007; Donders et al., 2008; Wojitani et al., 2012). These differences in genetic susceptibility can be attributed to genetic heterogeneity between different ethnicities and thus indicated the need, to evaluate these polymorphisms in different populations.

Of the seven commonly known haplotypes reported previously, only five haplotypes were observed in the present study (Madsen et al., 1995, 1998; Turner, 2003; Lee et al., 2005). The other two haplotypes HYPD and LYQC were not observed because codon 52 and 57 polymorphisms were not taken into consideration due to their low minor allele frequency. In addition to these well-known haplotypes, present study has also observed nine additional *MBL2* haplotypes, of which HXPA, LYQB, HYQB, LXQB, and HXQB, were observed with frequency ≥ 0.05. Out of these nine haplotypes, four haplotypes i.e., HXPA, LYQB, HYQA, LXPB were also reported previously (Verdu et al., 2006; Oudshoorn et al., 2008; Jha et al., 2014). The rest of five haplotypes are novel and reported for the first time in this study. These novel haplotypes were categorized into high and low secretor haplotypes based on the previous literature stating the association of wild allele of Y/X and A/B SNPs with high basal expression and variant alleles with low basal expression and hence circulating sMBL levels (Sumiya et al., 1991; Lipscombe et al., 1992; Madsen et al., 1994, 1995; Terai et al., 2003; Larsen et al., 2004; Garred et al., 2006; Antony et al., 2013; Jha et al., 2014; Das and Panda, 2015; Mashaly et al., 2016).

Stratification based on *MBL2* haplotypes has shown significantly high prevalence of HXPA haplotype in RVVI and MI cases, whereas, LXQB haplotype was significantly more common in RVVI cases. Thus, indicating X allele as an important marker providing risk toward RVVI. On the other hand, LYQB a low secretor haplotype provided significant protection against RVVI (OR; 0.58), BV (OR; 0.27), and MI cases (OR; 0.62) with high frequency observed in controls. However, the same haplotype was reported with a very low frequency (1%) in case of severe malaria in Indian population with no LYQB haplotype in control (Jha et al., 2014). Overall high prevalence of low secretor haplotypes were observed in both cases and controls of the present study. In agreement to these findings, the high prevalence of high secretor haplotype LYPA in cases and low secretor haplotype HYPD in controls was also reported previously in Brazilian population in Leprosy (de Messias-Reason et al., 2007). The high prevalence of low secretor *MBL2* haplotypes in different populations, including LYQC in Africans and LYPB in South American Amerindians was also documented (Boldt et al., 2006a,b). Taken together, the data suggests that sturdy forces have exerted positive selective pressure on these haplotypes across the globe.

Significantly low sMBL levels were observed in RVVI cases and its various categories as compared to controls, indicating MBL insufficiency as a possible risk factor for the rapid progression of RVVI. Similar to the situation of the present study, two studies have reported low MBL levels in cervicovaginal fluids of RVVC cases than controls (Babula et al., 2003; Liu et al., 2006). However, serum MBL analysis was preferred in the present study as vaginal secretion are reported to differs in MBL composition throughout the menstrual cycle as it derives both from plasma as well as from local synthesis. Furthermore, MBL level in vaginal secretions is also documented to be progesterone dependent (Bulla et al., 2010; Henić et al., 2010). The finding of the present study are contradictory to a study in Italian white Caucasian population where no significant difference was found when sMBL levels were compared between VVC, BV, and controls (Milanese et al., 2008) and to the study where significantly higher sMBL levels were found in women suffering from RVVC than in healthy women (Henić et al., 2010). Among various categories of RVVI cases, VVC cases showed significantly low sMBL levels as compared to BV and MI cases of the present study. MBL was shown to bind with diverse Candida species, inhibits growth and provides defense against fungal infections like VVC (Neth et al., 2000; Ip and Lau, 2004; Pellis et al., 2005; Moslem et al., 2015; Sobel, 2016). Hence, decreased sMBL levels observed in VVC cases of the present study reflects failure of defense mechanism and increased susceptibility in these cases. In addition to this, many of the bacteria and other pathogens have also been shown to be recognized by MBL (Neth et al., 2000; Townsend et al., 2001; Hamvas et al., 2005).

The present study also revealed an association of MBL polymorphism with its serum levels. It was found that for the same genotypes, MBL levels of cases were significantly low than respective control genotypes. As expected, sMBL concentration for A/B polymorphism in this study varied according to the individual's MBL genotypes with highest sMBL levels in wild genotype, and lowest in variant genotype (Madsen et al., 1995; Steffensen et al., 2000; Minchinton et al., 2002). This finding was in accordance with the study in Italian white Caucasian cases with recurrent vaginal infections (Milanese et al., 2008). Similar situation was also observed in cervicovaginal fluids of Latvian cases with recurrent VVC (Babula et al., 2003).

The sMBL levels were also studied in relation to high and low secretor haplotypes. Overall it was found that for the same haplotypes, sMBL levels of cases were contributing significantly low levels than control. However, when haplotypes were observed within cases and controls, it was found that LYPA “high secretor haplotype” was contributing significantly low MBL levels than LYQB “low secretor haplotype” in all cases types and control. Also, low secretor haplotype LXPA was contributing high sMBL levels in controls population while same haplotype LXPA was contributing low sMBL levels in cases. Similar results was also reported previously, where HYPA high secretor haplotype were significantly contributing low sMBL in the South American Indians than in other populations (e.g., the Eskimos; Madsen et al., 1998). These findings suggest the presence of some other down-regulating variants that exist outside the region of haplotype observed which cannot be omitted.

However, in the present study, allelic and genotypic frequencies of MBL polymorphisms deviated from HWE except that of control population in case of Y/X polymorphism (χ^2^ = 0.292; *p* = 0.589). Disagreement of genotypes distribution from HWE has been attributed to population stratifications or selection pressure (Hosking et al., 2004). Reason could be Infectious diseases or involvement of subjects from similar geographical area due to hospital based study (Miller, 1999; Ghosh, 2008; Fumagalli et al., 2009).

## Conclusions and future directions

The findings of the present study suggested low sMBL levels and X allele as contributing risk factors toward development of RVVI. Fortuitously, infusion therapy of recombinant MBL is indicated to be safe in MBL-deficient subjects (Valdimarsson et al., 1998; Garred et al., 2002). Thus, women with RVVI can be tested for the above-mentioned associations and might be benefitted from MBL replacement therapy. The genotype-phenotype relationship observed in the present study suggests RVVI and its types as multifactorial phenotypes, possibly implicating the combined effect of several host genes involved in innate immunity. Furthermore, the findings of this study also suggest the positive selection pressure exerted by RVVI, leading to increased frequency of novel low secretor haplotypes. Thus, further investigations involving detection of pathogen specific antibodies levels, *MBL2* expression analysis etc. are required to fully reveal the genetic, immunologic, and environmental variables that influence a woman's likelihood to develop RVVI.

## Author contributions

NK reviewed the literature, was involved in design, performing experiments, analysis, interpretation, and drafted the manuscript. MK, JS, SS, HA participated in the data analysis, manuscript editing and supervision. All authors read and approved the final manuscript.

### Conflict of interest statement

The authors declare that the research was conducted in the absence of any commercial or financial relationships that could be construed as a potential conflict of interest.
